# Our Medical Schools' Reopening

**Published:** 1920-10-09

**Authors:** 


### THE NEW SESSION.

### Our Medical Schools' Reopening.

During the past week introductory addresses and the usual October dinners have marked the re-opening of the great medical schools. It is difficult, perhaps, to speak in complete agreement with the theory propounded by the medical correspondent of the premier English daily, that any medical student who has started his school courses during the past week, is doing so at a moment lying definitely between two great medical eras. The beginner may be so placed from the practical teaching standpoint, but we would prefer to claim the early days of the past war as the more exact placing of the great transition period in medical thought.

Not only the problems concerned in maintaining men on the active list, but also the necessity for determining on a large scale the precise amount of endurance of which any particular human system was capable, made it imperative that the "beginnings of disease" should be accurately and rapidly noted. Thus thousands of medical men, young and old, found the whole outlook of their work and the type of professional opinions they had to give, completely revolutionised.

### An Obvious Difference.

Obviously, disease, from the military aspect is fundamentally different from that encountered in a peace-time civilian community, and its "beginnings" have corresponding variations, but the principles are and will remain the same in both. Even if the germ of the idea of preventive medicine, and the obvious reforms which it calls for, were there beforehand, we still owe much to the enormous impetus which the world war gave to medicine as applied to the masses. Our new medical teaching arises directly from the lessons then learnt and from the creditable skill with which our academic authorities have rapidly welded them into a practical and workable series of schemes.

Broadly speaking, the plans of each school are identical, if we allow for the modifications which relative availability of funds and accommodation make, and a description of the situation crystallises into the fact that a student is to be taught essential medicine, taught at the same time to teach himself, and, above all, trained to be capable of really caring for the people's health. To produce a good doctor it is quite unnecessary to insist that the student has a great technical knowledge of half-a-dozen "ancillary sciences," as we see them called. Even were it required, the ideal would be quite impossible. Each of these sciences has grown and is growing at a rate so remarkable that it is utterly impossible for the average man to grasp the details of even a-half of them to perfection. There are certain outstanding points, truths, and medical adaptations of each, the meaning of which he must appreciate and apply, but, as we all know by sad experience, the teaching methods of the past were chiefly remarkable for the insidious fashion in which these essentials were obscured by a cloud of matter, learned only to be forgotten the next year. How many times have we heard that a man only began to learn medicine *after* he was "qualified"?

### The Practical Doctor.

And so from October 1920 onwards all this is to be reformed. It is not only that the student is first to study health, and, later, disease; or that he is to be conducted to meet disease in its earliest manifestations in the human body. Rather will he now benefit by a system which brings to his notice many subjects ("public health" we used to call it), formerly quite neglected, teaches him ancillary sciences, but in their true medical bearing only, and, above all, makes him a practical doctor instead of an expert examinee.

There seems to be little doubt that the clinical unit system which is calculated to produce this type of training is a valuable innovation and likely to have a lasting application in our schools, but in teaching circles there is one other great consideration to be made. Examining bodies must see that their requirements also are adjusted to the best ideals of national health. On every hand we read the stated determination that this shall occur, and it remains now only to see that the examiners, who are, in some cases at least, men of the older school, shall individually put into practice the great lessons which the times have taught us.

